# Supplementary material for: Precise coupling of the thalamic head-direction system to hippocampal ripples
Source: Nat Commun. 2020 May 20;11:2524. doi: 10.1038/s41467-020-15842-4 (PMC7239903; doi:10.1038/s41467-020-15842-4)
Supplement: Supplementary file 1 — Supplementary Information [file 41467_2020_15842_MOESM1_ESM.pdf]

Precise coupling of the thalamic head-direction system to hippocampal ripples

Guillaume Viejo[1] and Adrien Peyrache[1,*]

[1] *Montreal Neurological Institute, McGill University, Montreal, QC, Canada;*
[*] *Corresponding author: adrien.peyrache@mcgill.ca*

Supplemental information

Supplementary Figures 1-3

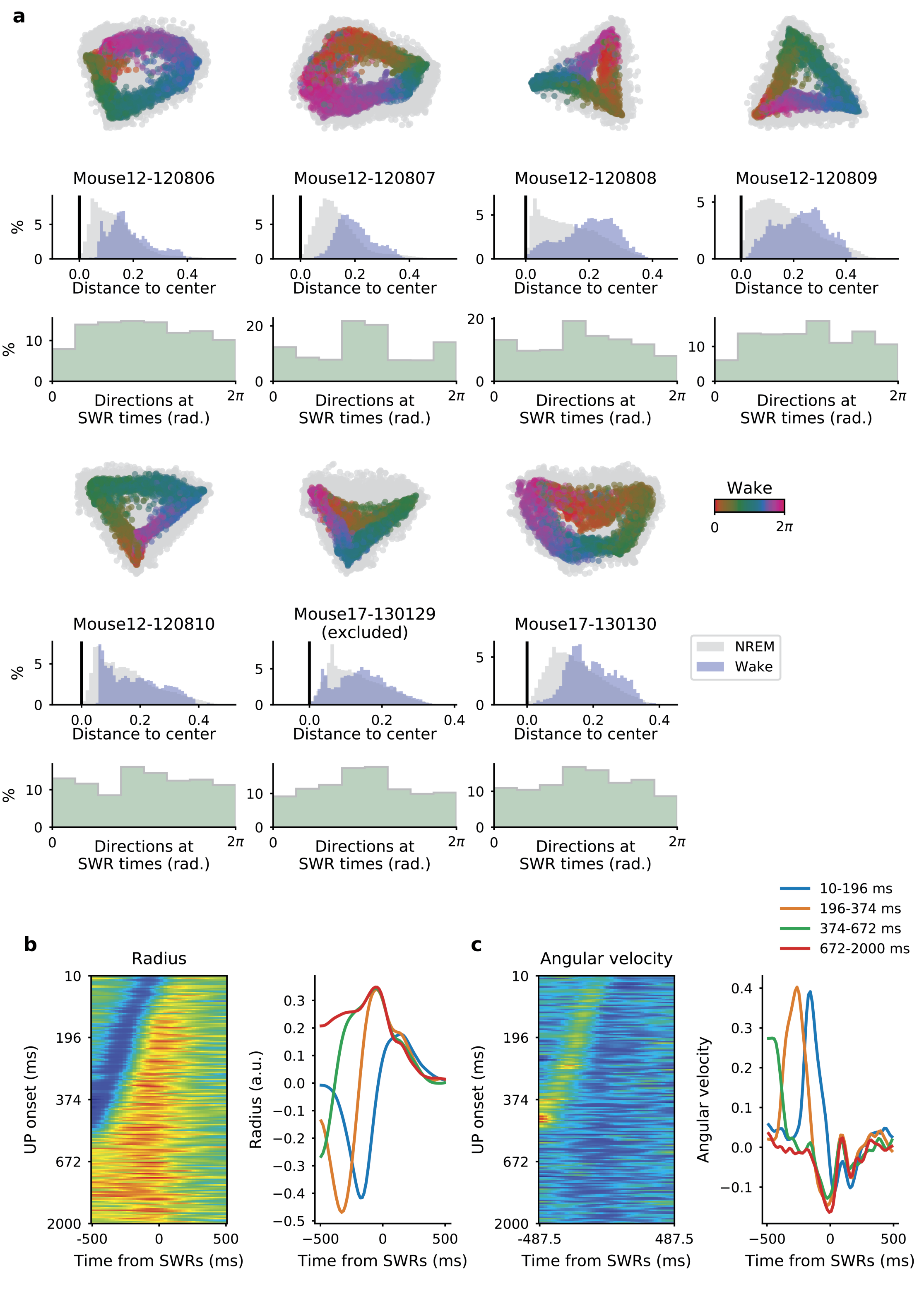

Supplementary Figure 1: ISOMAP embeddings and decoding. (**a**) ISOMAP embeddings (*top*), distance from center (*middle*) and average decoded direction at time of SWRs (*bottom*) for sessions with at least 10 HD neurons (same as **Fig.1c-e**). One session was excluded (Mouse17-130129) as the distribution of distances from center was shifted towards 0 (i.e. no ring topology). (**b**), *left* Average normalized radii around times of SWRs as a function of the time-lag from UP onset. (*right*) Average ISOMAP radius around SWRs occurring at various time intervals during the UP state (each of the four groups contains the same number of SWRs) (**c**) Same as (**b**) for angular velocity.

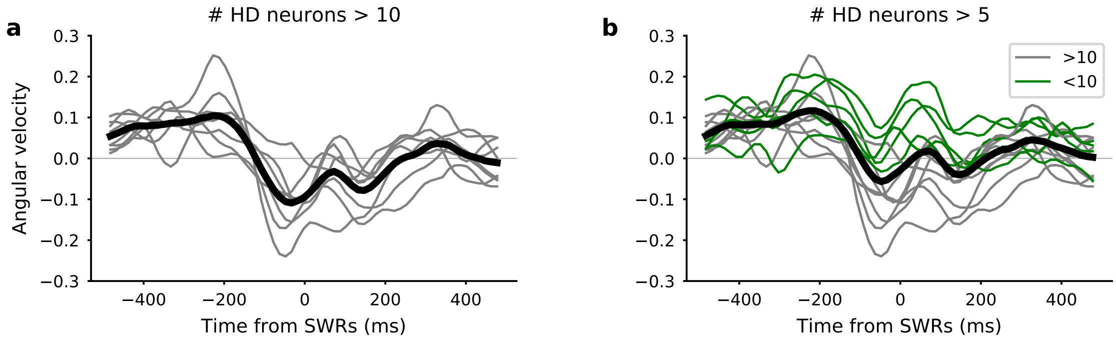

Supplementary Figure 2: Bayesian decoding during SWRs. (**a**) Angular velocity around SWRs using bayesian decoding for sessions with more than 10 HD neurons. Black line shows average. (**b**) Same as **a** for sessions with more than 5 HD neurons.

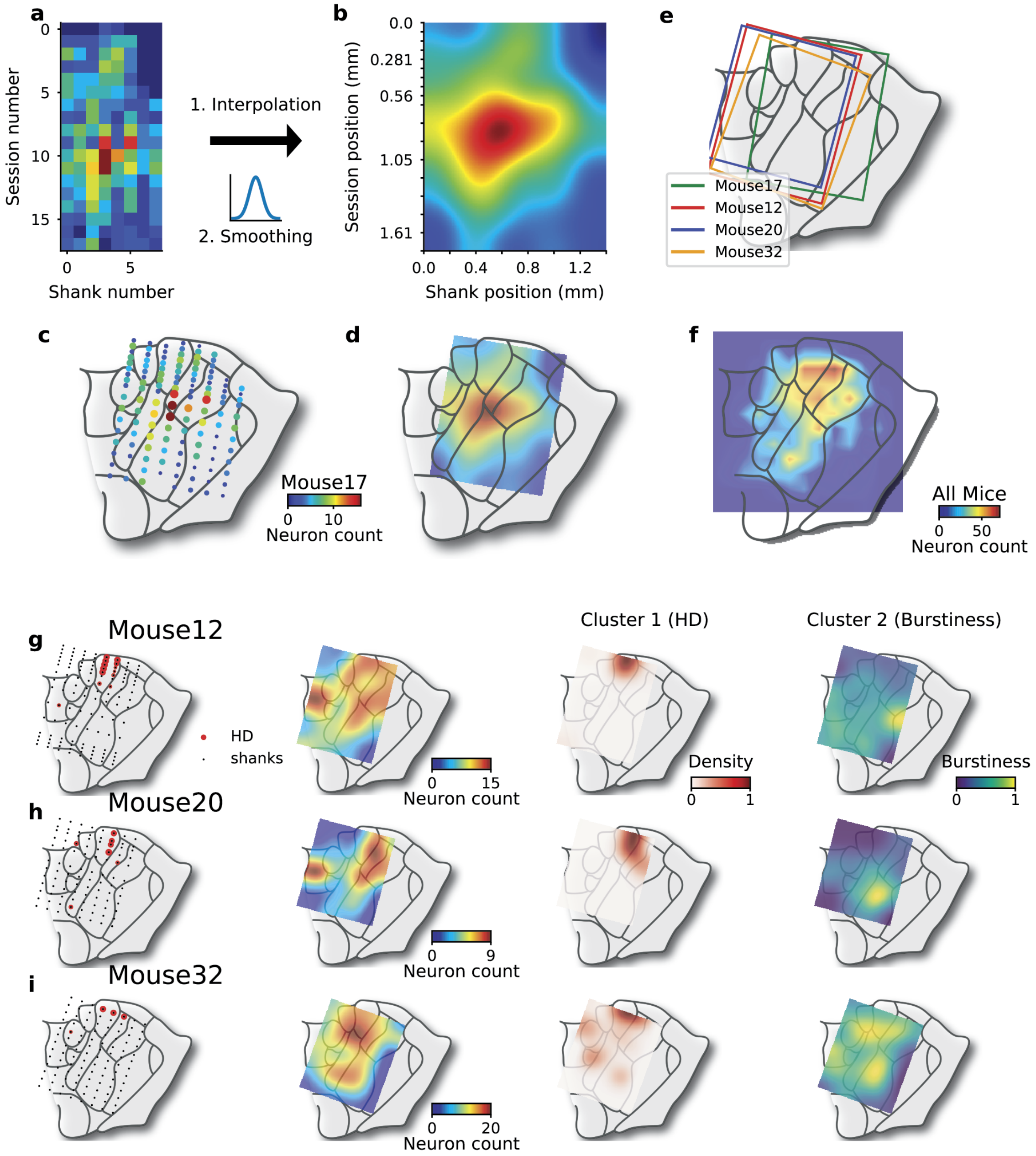

Supplementary Figure 3: Alignment of Recordings with Anatomical Locations. (**a**) Neuron count for each session and each shank for mouse 17. (**b**) Neuron density for mouse 17 after interpolation (i.e. given the distance between shanks and between sessions) and spatial smoothing. (**c**) Neuron count for each recording position. (**d**) Position of the interpolated neuronal density map (as in **b**). (**e**) Recording areas for each mouse. (**f**) Neuron count for all mice. (**g** - **i**) Recording locations, neuron count, cluster #1 (HD) and cluster #2 (burstiness) density maps (see Figure 5) for mice 12, 20 and 32.
